# Why Do People Not Attend for Treatment for Trachomatous Trichiasis in Ethiopia? A Study of Barriers to Surgery

**DOI:** 10.1371/journal.pntd.0001766

**Published:** 2012-08-28

**Authors:** Saul N. Rajak, Esmael Habtamu, Helen A. Weiss, Amir Bedri, Mulat Zerihun, Teshome Gebre, Clare E. Gilbert, Paul M. Emerson, Matthew J. Burton

**Affiliations:** 1 The London School of Hygiene and Tropical Medicine, London, United Kingdom; 2 The Carter Center, Bahir Dar, Ethiopia; 3 The Carter Center, Atlanta, Georgia, United States of America; 4 Light For The World, Addis Ababa, Ethiopia; University of Cambridge, United Kingdom

## Abstract

**Background:**

Trachomatous trichiasis (TT) surgery is provided free or subsidised in most trachoma endemic settings. However, only 18–66% of TT patients attend for surgery. This study analyses barriers to attendance among TT patients in Ethiopia, the country with the highest prevalence of TT in the world.

**Methodology/Principal Findings:**

Participants with previously un-operated TT were recruited at 17 surgical outreach campaigns in Amhara Region, Ethiopia. An interview was conducted to ascertain why they had not attended for surgery previously. A trachoma eye examination was performed by an ophthalmologist. 2591 consecutive individuals were interviewed. The most frequently cited barriers to previous attendance for surgery were lack of time (45.3%), financial constraints (42.9%) and lack of an escort (35.5% in females, 19.6% in males). Women were more likely to report a fear of surgery (7.7% vs 3.2%, p<0.001) or be unaware of how to access services (4.5% vs 1.0% p<0.001); men were more frequently asymptomatic (19.6% vs 10.1%, p<0.001). Women were also less likely to have been previously offered TT surgery than men (OR = 0.70, 95%CI 0.53–0.94).

**Conclusions/Significance:**

The major barriers to accessing surgery from the patients' perspective are the direct and indirect costs of surgery. These can to a large extent be reduced or overcome through the provision of free or low cost surgery at the community level.

**Trial Registration:**

ClinicalTrials.gov NCT00522860 and NCT00522912

## Author Summary

Trachoma is the commonest infectious cause of blindness worldwide. It causes trichiasis (inturning of the eyelashes to touch the eye), which can cause visual loss. Trachomatous trichiasis (TT) affects over eight million people, of whom 1.2 million live in Ethiopia – the most affected country worldwide. Surgery is the mainstay of treatment for TT. Despite the provision of free surgery in many areas, attendance rates are frequently low. An understanding of these barriers is fundamental for instituting measures to increase surgical uptake. In this study we interviewed 2591 people with TT in Ethiopia about their reasons for not having previously attended for surgery. The major barriers to attendance were lack of time, financial constraints and lack of an escort. TT is more prevalent in women and the elderly. Some barriers were particularly frequent in these high risk groups: lack of escort, fear of surgery and not knowing the surgical location impeded women more frequently than men, while financial constraints, transport difficulties and lack of escort affected more of those aged over fifty years than younger participants. It is likely that providing surgery closer to the patients would lessen many of these barriers.

## Introduction

Trachoma is the leading infectious cause of blindness worldwide [1]. The burden of disease falls largely on poor rural communities in Africa and Asia. Trachoma starts in childhood with recurrent infection of the tarsal conjunctiva by *Chlamydia trachomatis*, producing chronic inflammation (active trachoma). Tarsal scarring gradually develops, leading to entropion, trichiasis (in turning of eyelashes) and blinding corneal opacification. Trichiasis and corneal scarring usually develop in adulthood, although in high prevalence countries like Ethiopia, they can be found in children [2], [3].

The prevalence of active trachoma is decreasing, due to the combined impact of mass antibiotic treatment campaigns, environmental improvements and socio-economic development in endemic regions [1]. In contrast, there is less evidence for a decline in the number of people with trachomatous trichiasis (TT), which was estimated at 10 million worldwide in 1991 and 8.2 million in 2008 [1], [4], [5]. The World Health Organization-led Global Alliance for the Elimination of Blinding Trachoma by 2020 (GET 2020) has set an Ultimate Intervention Goal (UIG) for trichiasis of less than one known case of TT per 1000 total population [5], [6]. It is probable that at current rates of activity this target will not be met [7].

Trachomatous trichiasis is usually treated surgically. In the two most commonly used procedures, a horizontal incision is made either full thickness (bilamellar tarsal rotation) or partial thickness (posterior lamellar tarsal rotation) through the upper lid and sutures then place to rotate the lower part of the upper lid outwards [8]. However, surgical provision has generally been insufficient [9]. To address this, many countries, including Ethiopia, have made considerable efforts to scale-up surgical services in recent years. Unfortunately, despite this increased provision, the number of cases operated has grown less than anticipated. This is due to a range of service- and patient-specific barriers. The productivity of individual health care workers trained to perform TT surgery varies widely, but is often low [10], [11]. Retention of trained staff in rural settings is a major challenge and the availability of supplies and equipment can be erratic [10]. Even where services are reliably available uptake has been reported to be low in several countries (ranging between 18% and 66% of identified cases) [12]–[18].

Several studies have investigated why people with TT do not access treatment for this problem [12]–[15], [19]. A variety of reasons have been reported, including logistical/transport difficulties, financial constraints, and concerns about the success of surgery. Interviews with nurses providing TT surgery in Amhara thought patient-specific barriers to be the major reason for low surgical activity [10]. Some barriers can be partially overcome and higher uptake achieved by providing free surgery at the community level [16], [19]. However, even then uptake of surgery remains surprisingly low. This may reflect the indirect costs of receiving treatment: lost employment, additional childcare and accommodation and food near the surgical provider [20].

Over a million people in Ethiopia were estimated to have TT from surveys conducted in 2006 [1], [21]. In some areas, such as West Gojjam Zone in Amhara region (where the present study was conducted), 10% of the adult population were estimated to have TT [22]. Since 2001 TT surgery has been provided by the Amhara Regional Trachoma Control Programme. By 2008, when this study commenced, 404 health workers (mostly nurses) had been trained to perform TT surgery in Amhara Region, including 57 in West Gojjam Zone. These individuals are usually stationed in larger health centres where they may perform TT surgery alongside their other duties. In addition, surgical outreach campaigns are periodically conducted. However, despite these efforts, surgical activity has been relatively low in comparison to the need, at around 125,000 patients operated between 2001 and 2008; there are estimated to be over 600,000 people with TT in the Amhara region [22].

It is important to understand barriers to the uptake of surgery, so that strategies can be put in place to overcome them. In 2008, a series of surgical campaigns were conducted in rural locations in the West Gojjam zone to recruit participants to two clinical trials on the management of TT [23], [24]. This provided an opportunity to interview a very large number of individuals with unoperated TT concerning why they had not undergone surgery in the past.

## Materials and Methods

### Ethical permission

The study was approved by the National Health Research Ethics Review Committee of the Ethiopian Ministry of Science and Technology, the London School of Hygiene and Tropical Medicine Ethics Committee and the Emory University Institutional Review Board. All subjects provided informed consent. The informed consent was written for every subject. The research adhered to the tenets of The Declaration of Helsinki.

### Study participants

Individuals with previously un-operated TT (defined as one or more lashes touching the globe or evidence of epilation) were identified through a series of 17 surgical outreach campaigns in six districts (woredas) in the West Gojjam zone of the Amhara National Regional State, Ethiopia. The campaigns were advertised in local markets, churches and schools. Additionally, health extension workers, who are present in every sub-district (kebele) in Ethiopia, were trained to recognise trichiasis and visited homes and villages to identify affected individuals. All eligible patients attending with un-operated TT were recruited (no patients declined recruitment) and in some remote areas four-wheel drive vehicles were used to bring them for assessment at multiple health centres and rural clinics. Individuals were excluded from this study if they were pregnant (self reported), the trichiasis had been previously operated (i.e. was recurrent) or they were less than 18 years old. The vast majority of exclusions were patients who had received previous surgery. They were offered surgery (218 patients accepted, none decline) or in a few cases where there were only a few peripheral lashes that did not touch the cornea, provided with epilating forceps and advised to come to our follow-up. There were very few patients who were either pregnant or under 18 years old, although we did not count the precise number. Pregnant patients were offered a follow-up appointment after childbirth. Those under 18 who did not require a general anaesthetic were offered surgery and those who required a general anaesthetic were referred to the ophthalmologist at the local hospital.

### Assessment

**Figure 1:**
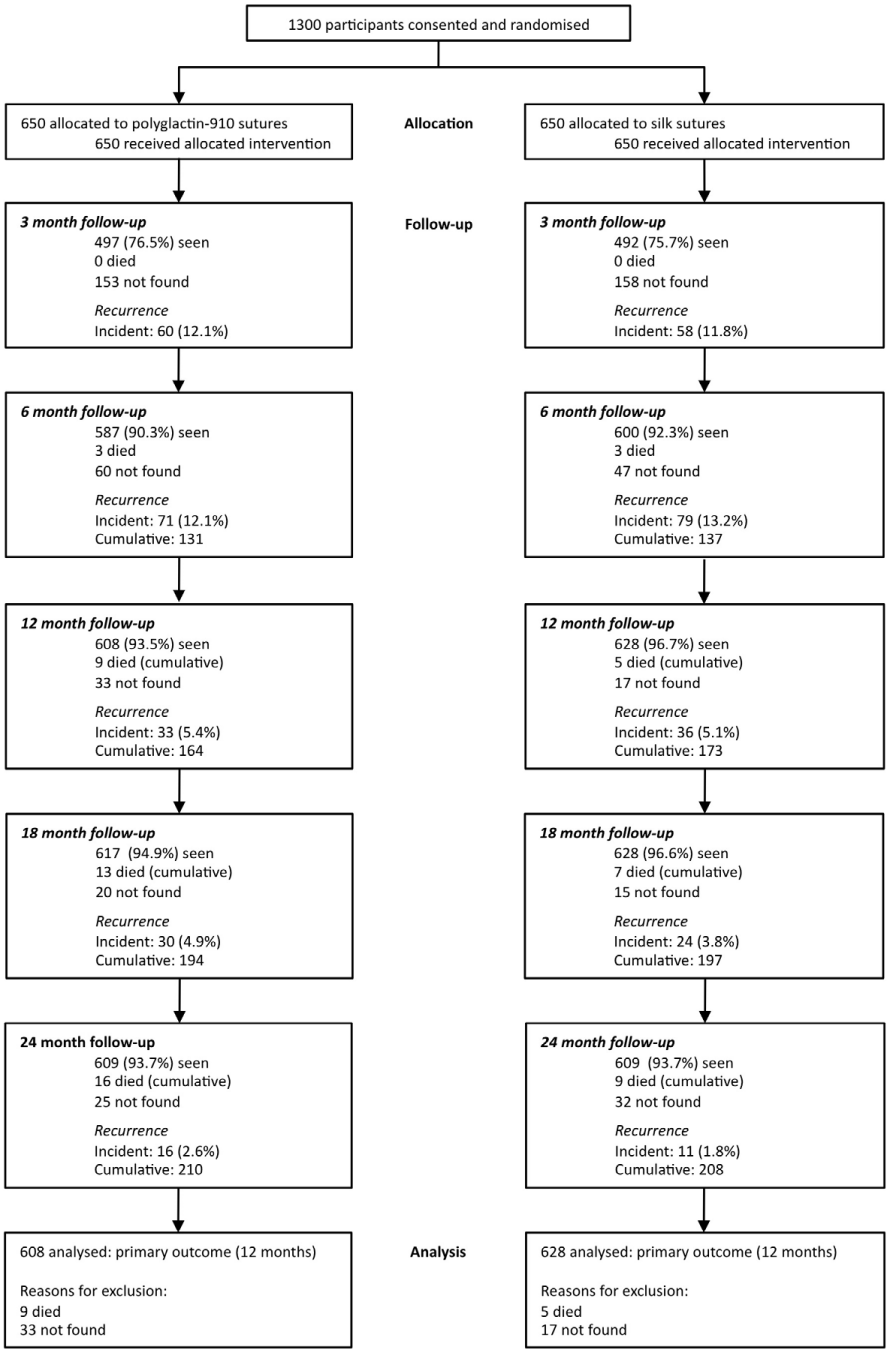
Consort flow chart 1. Absorbable versus silk sutures for surgical treatment of trachomatous trichiasis in ethiopia: a randomised controlled trial. PLoS Med 8: e1001137.

**Figure 2:**
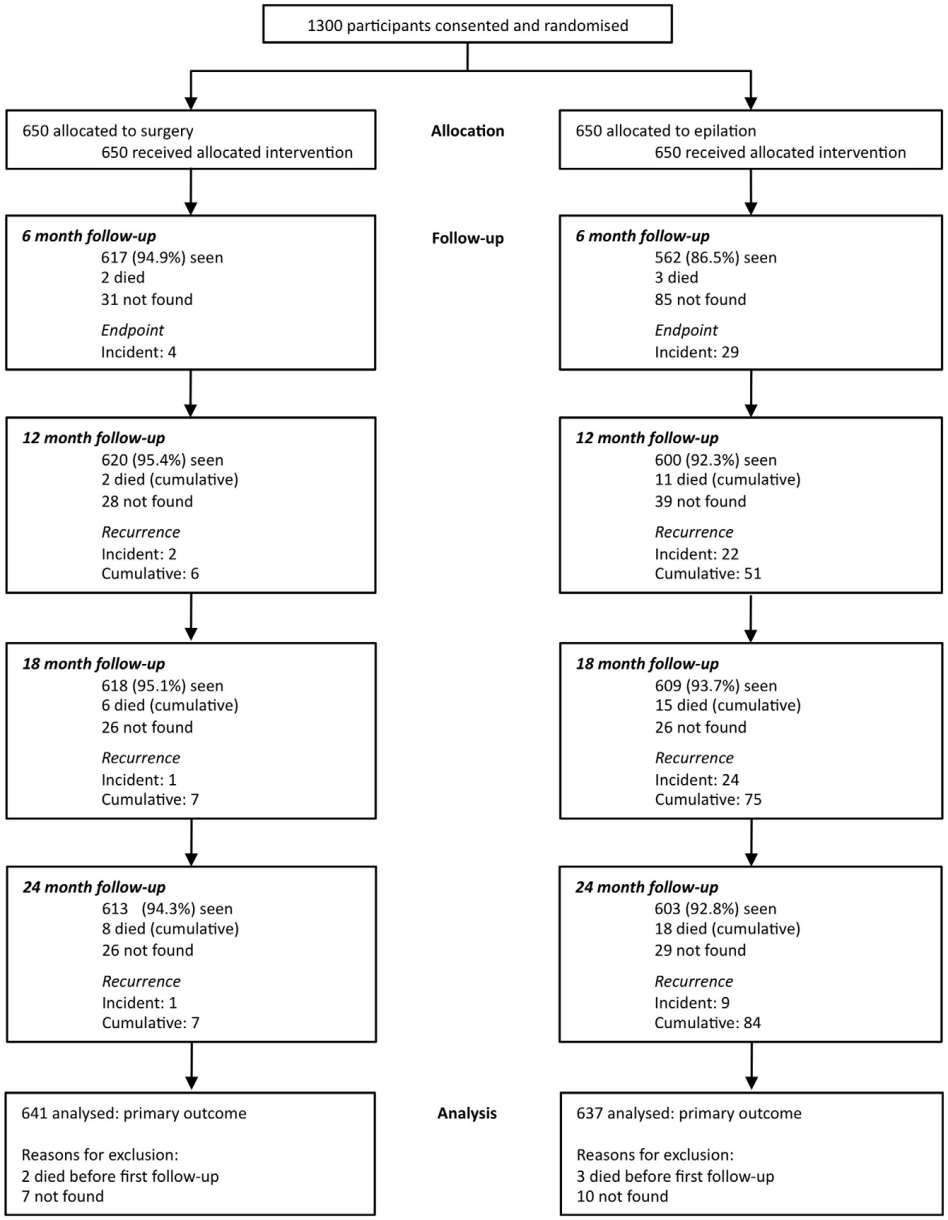
Consort flow chart 2. Surgery versus epilation for the treatment of minor trichiasis in ethiopia: a randomised controlled noninferiority trial. PLoS Med 8: e1001136.

Participants were assessed at campaign sites. These included health centres, clinics or vacant buildings, which were set up as temporary clinics. A questionnaire was administered in Amharic by trained local field workers. Participants were asked an open question regarding their reasons for not previously attending for TT surgery (‘barriers’). There was no limit to the number of barriers they could list, but if more than one reason was given, they were asked to state the foremost and second most important barrier. An ophthalmologist (SNR) examined the eyes for signs of trachoma, using a modification of the WHO (F,P,C) trachoma grading system to confirm the presence of trichiasis [25]. Trichiasis was defined as any eyelashes touching the eyeball or evidence of eyelash epilation. Some participants only had trichiasis affecting one eye, although data was recorded from both eyes. All participants were then enrolled into one of two clinical trials investigating the management of TT (http://ClinicalTrials.gov NCT00522860 and NCT00522912, fig. 1 and fig. 2) and received treatment for this free of charge [23], [24]. There were no individuals who were diagnosed with un-operated TT who declined enrolment onto the trials.

### Data analysis

Data were double entered into an MS Access database and analysed using STATA 9. The interview record form had a list of likely barriers that could be ticked and a free text box for other barriers. These barriers were all coded in the Access database..The frequency of specific barriers is reported and divided by whether the patient reported the barrier to be the primary or secondary obstacle. The primary and secondary barriers were combined and then divided by gender and age. The association between age and gender and specific barriers were analysed by logistic regression. This gender analysis was then adjusted for age and the age analysis by gender. A logistic regression model was developed to investigate whether there were age or gender specific differences in reported barriers previously being offered surgery.

## Results

We interviewed 2591 consecutive individuals presenting for trichiasis surgery. The clinical and demographic characteristics are presented in Table 1. All participants belonged to the Amhara ethnic group, with the exception of one (Tigray ethnic group). The majority (2406, 93%) were illiterate (98% of women and 78% of men). Amongst the 2591 participants there were 4387 eyes with TT; 793 (31%) people had unilateral disease and 1797 (69%) had bilateral disease. The disease severity varied from no lashes touching the eye (patients who successfully epilated) to 133 lashes touching (median 2, IQR:0–5).

**Table 1:** Demographic characteristics of participants and the clinical phenotype of all eyes.

Characteristic	Individuals		All Eyes	
	n/2591	(%)	n/5184	(%)
***Sex (female)***	1876	(72.4%)	-	-
***Age***				
18–29	244	(9.4%)	-	-
30–39	469	(18.1%)	-	-
40–49	650	(25.1%)	-	-
50–59	642	(24.8%)	-	-
60–69	437	(16.9%)	-	-
70+	149	(5.7%)	-	-
mean (sd, 95% C.I.)	50.0	(13.8, 49.5–50.6)	-	-
***Illiterate***	2406	(92.9%)	-	-
***Visual acuity***	***In better eye***		***All eyes***	
−0.2–0.3	612	(23.7%)	1710	(33.2%)
0.3–0.7	1017	(39.4%)	2037	(39.6%)
0.7–1.1	446	(17.3%)	728	(14.2%)
1.1–2.0	121	(4.7%)	191	(3.7%)
CF/HM/PL	304	(11.8%)	391	(7.6%)
NPL	81	(3.1%)	87	(1.7%)
Not measurable†	10		40	
***Corneal opacity grade*** *				
0	-	-	2176	(42.0)
1	-	-	1360	(26.2%)
2a	-	-	808	(15.6%)
2b	-	-	150	(2.9%)
2c	-	-	419	(8.1%)
2d	-	-	109	(2.1%)
3	-	-	132	(2.5%)
Phthisis	-	-	30	(0.6%)
***Trichiasis (number of lashes touching the eye)***				
None, no epilation†	-	-	790	(15.2%)
None, epilating	-	-	758	(14.6%)
1–5	-	-	2543	(49.1%)
6–9	-	-	551	(10.6%)
10–19	-	-	339	(6.6%)
20+	-	-	203	(3.9%)
***Entropion grade*** *				
0	-	-	1564	(30.2%)
1	-	-	1528	(29.5%)
2	-	-	1375	(26.5%)
3	-	-	402	(7.8%)
4	-	-	315	(6.1%)

* See protocol S1 for description of corneal scar and entropion grades.

† These eyes without TT were the second eye of patient's with unilateral disease.

Abbreviations:

CF: count fingers.

HM: hand movements.

PL: perception of light.

NPL: no perception of light.

VA: visual acuity.

The reasons individuals provided for not previously attending for TT treatment and the frequency with which each reason was reported as the first or second most important barriers are presented in Table 2. The three most frequent barriers were lack of time (45%), financial constraints (43%) and no-one available to accompany them (31%).

**Table 2:** Reasons for not previously having TT surgery.

Barrier	Overall Frequency†		Primary Barrier		Secondary Barrier‡	
	n/2591	(%)	n/2591	(%)	n/2591	(%)
Lack of time	1174	(45.3%)	677	(26.1%)	401	(15.5%)
Financial constraints	1112	(42.9%)	809	(31.2%)	246	(9.5%)
No-one to accompany	807	(31.2%)	349	(13.5%)	424	(16.4%)
No symptoms	330	(12.7%)	286	(11.0%)	32	(1.2%)
Fear of surgery	199	(7.7%)	161	(6.2%)	33	(1.3%)
Had symptoms, but did not know treatment needed	155	(6.0%)	136	(5.3%)	10	(0.4%)
Previously attended clinic but did not receive treatment	108	(4.2%)	87	(3.4%)	15	(0.6%)
Surgery location unknown	91	(3.5%)	18	(0.7%)	62	(2.4%)
Transport difficulties	72	(2.8%)	21	(0.8%)	36	(1.4%)
Poor health	28	(1.1%)	17	(0.7%)	10	(0.4%)
Resistance of other family members	20	(0.8%)	10	(0.4%)	9	(0.4%)
Used alternative treatment*	17	(0.7%)	16	(0.6%)	1	(0.04%)
Does not trust medical professionals	2	(0.1%)	2	(0.1%)	0	(0.0%)
Thinks surgery is a sin	2	(0.1%)	1	(0.04%)	1	(0.04%)
Thought condition was resolving without intervention	1	(0.04%)	1	(0.04%)	0	(0.0%)

The overall frequency of each reported barrier and the frequency it was reported as either the primary or secondary barrier.

* Alternative treatments used with expectation of curing TT:

Expected Azithromycin provided in mass drug administration campaign to treat trichiasis: 8 people.

Church/prayer: 4 people.

Unidentified medicine: 2 people.

Epilation: 3 people.

† >1 barrier cited by some study participants.

‡ 1311 participants did not cite a secondary barrier.

There were differences in barriers between men and women (Table 3). In multivariable modelling, women more frequently reported that no-one was available to accompany them, fear of surgery and not knowing the surgery location. Men reported fewer symptoms, had more frequently attended clinics but found surgery was unavailable, and had tried more alternative treatments. Lack of time and financial constraints were mentioned with equal frequency by men and women.

**Table 3:** Barriers to attending for surgery divided by gender.

	a) Frequency of barriers				b) Analysis of association between barrier and gender					
Barrier	Male		Female		Univariate Analysis			Analysis adjusted for age (≥50years)		
	n/715	(%)*	n/1876	(%)*	OR	95%CI	p-value	OR	95%CI	p-value
No symptoms	140	(19.6)	190	(10.1)	0.46	(0.36–0.59)	<0.001	0.39	(0.30–0.50)	<0.001
No-one to accompany	140	(19.6)	667	(35.5)	2.27	(1.84–2.79)	<0.001	2.45	(1.99–3.03)	<0.001
Fear of surgery	23	(3.2)	176	(7.7)	3.11	(2.00–4.85)	<0.001	3.15	(2.01–4.92)	<0.001
Surgery location unknown	7	(1.0)	84	(4.5)	4.74	(2.18–10.3)	<0.001	5.14	(2.36–11.2)	<0.001
Previously attended clinic but did not receive treatment	40	(5.6)	68	(3.6)	0.63	(0.43–0.95)	0.026	0.60	(0.40–0.90)	0.013
Alternative treatment tried	10	(1.4)	7	(0.4)	0.26	(0.10–0.70)	0.007	0.23	(0.09–0.63)	0.004
Lack of time	336	(47.0)	838	(44.7)	0.91	(0.77–1.08)	0.29	0.87	(0.73–1.04)	0.13
Financial constraints	309	(43.2)	803	(42.8)	0.98	(0.83–1.17)	0.85	1.05	(0.88–1.25)	0.59
Transport difficulties	18	(2.5)	54	(2.9)	1.15	(0.67–1.97)	0.62	1.36	(0.79–2.35)	0.27

Part a) All barriers cited by participants divided by gender. Part b) Analysis of association between being female and specific barriers.

* Denominator is number of men/women; numerator is number of men/women citing each barrier. For example 19.6% of men and 10.1% of women cited no symptoms as a barrier and.

There were differences in reported barriers between people greater or less than 50 years of age (Table 4). People 50 years or older were more frequently unable to attend surgery because of financial constraints, transport difficulties or a lack of someone to accompany them. Those of younger than 50 years more frequently reported that lack of time or symptoms as barriers.

**Table 4:** Barriers to attending for surgery divided by age (less or greater than 50 years).

	a) Frequency of barriers				b) Analysis of association between barrier and age					
Barrier	Age<50 years		Age≥50 years		Univariate Analysis			Multivariable Analysis		
	n/1586	(%)*	n/1909	(%)*	OR	95%CI	p-value	OR	95%CI	p-value
No symptoms	192	(16.1)	138	(9.9)	0.57	(0.45–0.72)	<0.001	0.47	(0.37–0.60)	<0.001
No-one to accompany	341	(28.5)	466	(33.4)	1.25	(1.06–1.48)	0.008	1.45	(1.22–1.72)	<0.001
Fear of surgery	96	(8.0)	103	(7.4)	0.91	(0.68–1.22)	0.53	1.05	(0.79–1.41)	0.730
Surgery location unknown	37	(3.1)	54	(3.9)	1.26	(0.82–1.93)	0.29	1.50	(0.98–2.31)	0.063
Previously attended clinic but did not receive treatment	55	(4.6)	53	(3.8)	0.82	(0.56–1.20)	0.31	0.74	(0.50–1.10)	0.14
Alternative treatment tried	9	(0.8)	8	(0.6)	0.76	(0.29–1.97)	0.57	0.57	(0.21–1.51)	0.26
Lack of time	570	(47.7)	604	(43.3)	0.84	(0.72–0.98)	0.024	0.82	(0.70–0.96)	0.013
Financial constraints	464	(38.8)	648	(46.4)	1.36	(1.17–1.60)	<0.001	1.38	(1.17–1.61)	<0.001
Transport difficulties	19	(1.6)	53	(3.8)	2.44	(1.44–4.15)	0.001	2.56	(1.50–4.38)	0.001

Part a) All barriers cited by participants divided by age less or greater than 50 years. Part b) Analysis of association between being ≥50years and specific barriers.

* Denominator is number of men/women; numerator is number of men/women citing each barrier. For example 16.1% of participants age<50 years and 9.9% of participants age≥50 years cited no symptoms as a barrier.

One hundred and fifteen (4.4%) individuals had been offered surgery previously. Women were less likely to have been offered surgery before; neither severe disease nor poor vision were associated with having previously been offered surgery (Table 5).

**Table 5:** Univariate and multivariable associations for having been previously offered TT surgery.

	Univariate Analysis			Multivariable Analysis		
Variable	OR	95% CI	p-value	OR	95% CI	p-value
Gender (female)	0.70	0.53–0.94	0.017	0.69	0.52–0.93	0.014
Age (>50 years)	0.90	0.68–1.18	0.45			
Illiterate	1.11	0.86–1.43	0.43			
Severe entropion*	1.33	0.93–1.92	0.12	1.36	0.95–1.96	0.095
Poor visual acuity†	0.91	0.67–1.25	0.57			

* Entropion grade 3 or worse.

† Visual acuity worse than logMAR 0.7.

## Discussion

To achieve the UIG of reducing “known” cases of TT to less that 1/1000 population by the year 2020 will require a marked increase in the amount of TT surgery performed [7]. Despite the scale-up of surgical services in recent years, current surgical activity is not effectively tackling the backlog. There are multiple provider- and patient-specific barriers. We have previously reported a study from the same area of Ethiopia of the determinants of TT surgeon retention and productivity, and an investigation of the major challenges to delivering a service from the provider's perspective [10]. Here we examined barriers from the patients' perspective.

Although participants mentioned many different barriers, the dominant ones relate to the varying direct and indirect costs surgery. Financial constraints were frequently cited. Prior to this free community outreach campaign, surgical provision in this area was mostly provided through government health centres in larger towns, which often charge a fee (US$5–8, average monthly income approximately $20), or by private clinics. In addition to direct fees for treatment there are often multiple indirect costs: transport, food, accommodation, childcare and lost employment. It is also possible that patients may have felt that the distance to government clinics that provided TT surgery was prohibitive. Older people reported financial constraints more frequently than younger people, perhaps reflecting more limited resources. The financial barrier to obtaining treatment has been consistently reported from other countries [13], [15], [18]. In Ethiopia, two studies have found indirect costs to be a barrier to TT surgery, with one of them showing this to be a greater impediment than the health centre fees [19], [20].

Participants frequently reported “lack of time” to be a barrier, which is consistent with other studies of barriers to surgery [12], [13], [15], [19]. Although, tarsal rotation surgery, as practised in Ethiopia, only takes about 20 minutes per lid, the whole process of accessing and recovering from lid surgery may take as much as a fortnight. Health centres that provide surgery can entail three days walk each way in this part of Ethiopia. After reaching the health centre it is not unusual to wait one or two days to be seen by the surgeon, and further days before the surgery is performed. Following discharge patients need to return for sutures to be removed 7–10 days post-operatively, if non-absorbable material is used. They may not be able to return to their usual activities for a week or two after the surgery. Furthermore, there is a common misconception that no work should be done for three months after surgery (unpublished data). Younger participants cited lack of time more frequently than older patients, probably because they have greater childcare responsibilities and are more likely to be the economically productive member of the family. Moreover, some participants may have felt comfortable citing ‘lack of time’ rather than admitting to a lack of financial resources.

TT is frequently bilateral and it is usual to operate on both eyes on the same day. After surgery, a pressure dressing is taped over the eye and the patient asked to keep this in place until the following morning. Therefore patients leave the operating theatre with both eyes occluded. They require a helper (friend/relative) to accompany them home and may need further assistance in the first few days after surgery. This was particularly a problem for women and older patients. Other studies have reported a similar barrier, indicating that this is a consistent problem for many people [13], [19], [20]. There are further indirect costs for the accompanying person in terms of time, accommodation, food and potential loss of earnings.

Only a few participants reported being unsure of where to go for treatment (3.5%) or that they did not require treatment (6%). This indicates that awareness and understanding of the condition is relatively high in this part of Ethiopia, despite the remote rural settings in which many of these patients live. Other studies have reported similar or slightly lower rates of awareness [13], [15], [20]. Therefore, overcoming the patient barriers to accessing surgery needs to largely focus on the direct and indirect cost of surgery.

The uptake of the two approaches to delivering surgery, i.e. at health facilities or through community-level outreach programmes, have been compared in a randomised trial in The Gambia, which showed attendance to be significantly higher with village-based surgery (66% attendance) than health centre based surgery (44% attendance) (rate ratio: 1.49, 95% C.I.:1.11–2.01, p = 0.009) [16]. Surgical campaigns in villages may be particularly effective at reaching larger numbers of patients in Ethiopia (although logistically challenging to deliver) as it is a large country with very limited road and transport infrastructure [10]. Moreover, a walking distance of greater than hour has been shown to be associated with decreased attendance for TT surgery in this region [19]. They are also likely to be particularly beneficial for reaching women and older people for whom transport, distance and lack of escort were particular barriers. In recent years there has been a trend towards strengthening integrated clinic-based services and reducing ‘vertical’ surgical campaigns. Recent data on the relative productivity of these two approaches clearly favours the use of outreach campaigns [10]. However to date, no work has been done to compare the outcomes of the two delivery models. The present study suggests that a locally delivered outreach campaign providing free or low-cost surgery would overcome or reduce the three most common barriers from the patents perspective.

Trichiasis encompasses a wide spectrum of disease from a single in-turned lash to complete entropion of the lid [26]. Symptoms vary, from mild ocular irritation to intense pain, photophobia and epiphora [27]. In this and previous studies some patients had only mild symptoms which they did not consider warranted surgery [12], [13], [15], [18], [19]. Men reported “no symptoms” more frequently than women. This may reflect prioritisation of work or farming over eye care.

Some patients (4%) reported that they had in fact attended a health facility for surgery but not received treatment. It was not possible to determine the reasons why the health facility had not provided surgery. Research in Ethiopia and Tanzania has found that trained surgeons are generally providing very little clinic-based surgery [10], [11]. In Ethiopia, surgeons reported multiple barriers to providing surgery including a lack of surgical supplies, conflicting work duties, little supervision and administrative support [10]. It is important that surgical programmes not only focus on training the surgeons and providing central suppliers with equipment, but also provide ongoing support and training to the individual surgeons, clinics and outreach campaigns, to ensure the correct equipment, consumables and well-trained personnel are available simultaneously when needed.

Cataract is the leading cause of blindness worldwide, which is also alleviated by surgery. Studies of barriers to receiving cataract surgery have been conducted in a wide range of resource poor settings and are strikingly similar to those reported for TT surgery [20], [28]–[40]. The vast majority of these have identified cost as the major barrier, although some have also found transport difficulties (and cost), lack of time, fear of surgery, lack of awareness of available treatment and lack of desire to improve vision to be secondary barriers. Direct and indirect costs may be a particular problem for cataract surgery provision, because cataract surgery has not been available free of charge in many settings and because the specialised and expensive equipment requirements restrict provision in the community.

A limitation of this study is that information was collected only from people who voluntarily attended the surgical campaigns. In order to minimise the potential bias in the sample of TT patients being studied, treatment campaigns were provided in multiple rural villages. Health extension workers conducted an extensive mobilisation campaign with door-to-door visits in villages and advertising in churches and markets, to encourage maximal uptake of the free service being offered in the community. The surgical campaigns were conducted in the non-farming season. However, it is likely that some TT patients did not present for treatment; these people may have different or more profound barriers to attending for surgery. A further limitation is the presence of an expatriate doctor at all of the campaigns. It is possible that this increased attendance at campaigns, therefore distorting the reasons for previous non-attendance. However, the barriers reported in this study are strikingly similar to those of other studies in which there is no indication of an expatriate being present [18], [19].

Studies of patient barriers to TT surgery uptake have previously been conducted in Malawi, Tanzania and The Gambia reporting strikingly similar barriers [12], [14], [15], [17]. Our study supports the findings of an earlier, smaller study in Ethiopia and indicates the importance of reducing indirect costs to the patient when providing TT surgical services [19]. It also highlights the need to target women and the elderly for whom the burden of disease and barriers to accessing services are greater. These barriers could be significantly reduced through the delivery of low cost or free surgery within the community, perhaps through small mobile surgical teams, although the relative efficacy and cost of this delivery method requires evaluation.

## Supporting Information

Protocol S1 **Trial protocol for: Absorbable versus silk sutures for surgical treatment of trachomatous trichiasis in ethiopia: a randomised controlled trial.** PLoS Med 8: e1001137.
(DOC)

Protocol S2 **Trial protocol for: Surgery versus epilation for the treatment of minor trichiasis in ethiopia: a randomised controlled noninferiority trial.** PLoS Med 8: e1001136.
(DOC)

Checklist S1 **Consort checklist for: Absorbable versus silk sutures for surgical treatment of trachomatous trichiasis in ethiopia: a randomised controlled trial.** PLoS Med 8: e1001137.
(DOC)

Checklist S2 **Consort checklist for: Surgery versus epilation for the treatment of minor trichiasis in ethiopia: a randomised controlled noninferiority trial.** PLoS Med 8: e1001136.
(DOC)
