# Supplementary material for: Why Do People Not Attend for Treatment for Trachomatous Trichiasis in Ethiopia? A Study of Barriers to Surgery
Source: PLoS Negl Trop Dis. 2012 Aug 28;6(8):e1766. doi: 10.1371/journal.pntd.0001766 (PMC3429389; doi:10.1371/journal.pntd.0001766)
Supplement: Protocol S1 — Trial protocol for: Absorbable versus silk sutures for surgical treatment of trachomatous trichiasis in ethiopia: a randomised controlled trial. PLoS Med 8: e1001137. (DOC) [file pntd.0001766.s001.doc]

**Strategies for the management of trachomatous trichiasis**

**Trial 1: Randomised controlled trial of vicryl sutures for trachoma surgery.**

**Study Protocol**

**Summary**

Trachoma is the leading infectious cause of blindness worldwide. Chronic keratoconjunctivitis caused by Chlamydia trachomatis causes scarring of the inner eyelid. This in turn causes in-turning of the eyelid (entropion), causing the eyelashes to turn inwards (trichiasis) and rub the cornea, resulting in scarring and visual impediment. SAFE is the WHO initiative for the control of trachoma. It comprises, Surgery, Antibiotics, Facial cleanliness and Environmental improvement. The surgery aims to correct the trichiasis, thereby preventing further visual loss. However it is known that trichiasis returns in as many as 50% of people who have had surgery. This trial aims to determine the best suture for trachoma surgery, in order to try and reduce the recurrence.

Specific aims:

1) To determine if the recurrence of trichiasis, progression of conjunctival scarring, progression of corneal scarring and progression of visual loss are less with absorbable (vicryl) or non-absorbable sutures (silk)

2) To determine if there are factors other than recurrence of trichiasis that contribute to progression of scarring, opacity and visual loss, including:

- Concurrent bacterial infection
- Dry eye

**Trial Location and format**

The trials are planned to be undertaken in the West Gojjam zone, Amhara.

Trachomatous trichiasis surgical campaigns will be organised in several health centres and/or health posts. Recruitment will take place during these campaigns.

**A: Trial Recruitment Phase (see diagram 1 below)**

1. **Identify locations (see diagram 2 below) for different parts of recruitment process**

Team Members who will perform this (appendix A)

SR (research fellow) and nurse 1 (examination nurse)

1. **Presentation to patients and accompanying people**

Team Members

Assistants 1 and 2 (consent field workers)

Procedure

- 1. Hand out project information sheets.
  2. Introduction: who we are.
  3. Info about what is known about trachoma.
  4. Info about what is not known about trachoma.
  5. Info about the trials.
  6. Info about safety to patient.
  7. Info about what we will do from now.

Late comers will receive similar presentation individually or in small groups.

1. **Field worker to call SR or EM to triage Patient to:**
   1. Trial 1 or trial 2
   2. Treatment for right eye/left eye/bilateral
   3. Decide which is trial eye if bilateral TT

In unilateral cases the study eye is the one with major trichiasis.

In bilateral major trichiasis cases randomly select the eye which will be followed in the study by using the left/right random generator table. Both eyes receive the same suture type at the time of surgery.

Procedure

1. Patients with trachomatous eye disease and no exclusion criteria, enter into study.
2. Patients with trachomatous eye disease and exclusion criteria. Do not enter into study, but give standard trachoma treatment, including surgery if required.
3. Patients with non-trachomatous eye disease:
   - 1. Emergency: to be seen by ophthalmologist or ophthalmic nurse and treated if necessary or arrangements made for urgent referral to local eye facility
     2. Non-emergency: to be seen by ophthalmic nurse and instructions for attendance at local eye unit with referral letter if required.

Inclusion criteria – trial 1

Patients with major trachomatous trichiasis (greater than 5 eyelashes touching the globe of either or both eyes), who consent to be part of the trial and do not have any of the exclusion criteria below.

Inclusion criteria – trial 2

Patients with minor trachomatous trichiasis (5 or less eyelashes touching the globe of either or both eyes), who consent to be part of the trial and do not have any of the exclusion criteria below.

Exclusion criteria for both trials

- Age less than 18 years
- Previous eyelid surgery
- Medically unfit, BP systolic >170, diastolic >110.
- Pregnant (self reported, or clearly evident)

1. **Consent**

Team Members

Assistants 1 and 2 (Consent field workers)

Procedure

- 1. Check if they heard initial lecture and if so whether they understood it.
  2. If not, then explain trachoma/trial.
  3. Check if they have received the information leaflet and had time to read it. If not received then give this to patient and time to read.
  4. If patient is illiterate, information to be read to them by trained assistant.

Procedure to follow if patient does not consent or is not competent to consent

- Becomes non-trial patient.
- Continue as per local protocol, ensuring that patient is still offered surgery by the project team.

Identification of which trial each patient is in

Attempt to sit patients being recruited into the two different trials in different waiting areas. Give patient large piece of card with trial number and eye to be treated (right/left/bilateral) written on it.

1. **Questionnaire (see appendix B)**

Team Members

Assistants 1 and 2 (Consent field workers)

Contents

Part 1: demographics

Part 2 (part 3 of the study record form, appendix B): symptomatology, surgical and epilation history

1. **Visual acuity, height and weight**

Team Members

Assistants 3 and 4 (Vital statistics field workers)

Procedure

1. Logmar 4m tumbling E chart
2. Height
3. Weight
4. **Examine and photograph patient**

Team Members

SR, assistant 5 (examination field worker) and field co-ordinator when triaging finished

Procedure

See appendix B

1. **Randomise patient to one of the two arms of appropriate trial for that patient (appendix C)**

Team Members

Assistant 6 (randomisation field worker)

Procedure

The full randomisation procedure is described in appendix C.

The part of this describing the ‘in field’ procedure is described below. This will be performed by a dedicated randomisation field worker.

1. Randomisation field worker will be working in location not visible to other project team members.
2. After consent and clinical examination the patient is sat in a waiting area.
3. When a surgeon is available for operating, the surgeon finds the randomisation field worker and together they take the patient through to the theatre.
4. Again, together they take the next envelope from that surgeon’s pile and open it. They will open the envelope and record the instruction on two copies of masterlist S2 (see below). The first copy will be kept by the randomisation field worker and the second copy being given to a person independent of the trial. A new masterlist S2 sheet will be used on each trial day.
5. Field worker will:
   1. Take 3 (if unilateral surgery required) or 6 (if bilateral) of the correct sutures from the suture store and give to the surgeon. They will ask the surgeon to double check that this is the correct suture.
   2. They will use masterlist S3 (see below) to record whether the patient needs suture removal to be performed in 7-10 days. This will be recorded on two copies of masterlist S3.

Master list S2

| Date | Study Number | Name | Randomisation Envelope | Study Eye | Sutures Used |
| --- | --- | --- | --- | --- | --- |
|  | 1 | XX | S1/0001 |  | V |
|  | 2 | Xx | S2/0001 |  | V |
|  | 3 | Xx | S3/0001 |  | S |
|  | 4 | Xx | S1/0002 |  | V |
|  | 5 | Xx | S3/0002 |  | S |
|  | 6 | Xx | S2/0002 |  | S |

Masterlist S3 – suture removal

| Unique identifier | Name | Date of surgery | Date for suture removal | Eye for suture removal (R/L/B) | Sutures successfully removed  (yes or no) |
| --- | --- | --- | --- | --- | --- |
|  |  |  |  |  |  |
|  |  |  |  |  |  |
|  |  |  |  |  |  |

1. **Suture and surgery instructions:**

Team Members

Nurses 2, 3 and 4 (surgical nurses), assistant 7 (surgical assistant)

Procedure

- 1. Nurse 2, 3 or 4 to operate on patient
  2. Nurse 2, 3 or 4 will record the surgical outcome on masterlist S4 (see below)
  3. Nurse 2, 3 or 4 to give post-op instructions
     1. Tetracycline
     2. Keep eye clean
     3. Not to rub eye
     4. Return 7-10 days for removal of silk (BLACK) sutures and recording of immediate post-op complications

The Trabut surgical technique will be used (appendix D)

Sterilization will be as per The Trichiasis Surgery for Trachoma WHO manual (appendix E)

All sharps (needles, blades etc) will be disposed of in specialised ‘sharps bins’

Masterlist S4

| Unique identifier | Name | Date of surgery | Surgery routine or complicated | Complication (if applicable) |
| --- | --- | --- | --- | --- |
|  |  |  |  |  |
|  |  |  |  |  |
|  |  |  |  |  |

Procedure to follow if intra-operative complication

- Follow local policy
- However, ensure that same suture as was initially randomised is still used, even if more or less than usual are needed.
- Masterlist S4 (see above) will be used to record any intra-operative complications.

1. **Recruitment of controls**

When

Between or after surgical campaigns

Who

People without trichiasis or tarsal conjunctival scarring. Need to be matched for:

1. Age within 10 years
2. Same sex
3. Same village
4. Recruited within 3 weeks of matched case.

Procedure

Information sheet

Consent

Study form

Examination, photos and swabs (as above)

**B: Trial Follow-up Phase**

1. **7-10 day follow-up**

Team members

Nurse 5

Driver 2, if patient fails to attend for follow up

- 1. Trial 1
     1. Find appropriate patients according to suture removal lists from each trial
     2. Record post operative examination on new form for patient
     3. Remove BLACK sutures only.
     4. Record removal of sutures on masterlist S5
     5. Give patient further instructions

Masterlist S5

| Unique identifier | Name | Date of suture removal | Eye of suture removal | Early post operative findings |
| --- | --- | --- | --- | --- |
|  |  |  |  |  |
|  |  |  |  |  |
|  |  |  |  |  |

Examination

1. Are/is there:
   1. Discharge
      1. Purulent
      2. Non-purulent
   2. Obvious granuloma (do NOT evert lid)
   3. Bleeding
   4. Early recurrence
      1. Count eyelashes and record
   5. Skin infection / cellulitis
2. Is further surgery being planned?
   1. When?
   2. Where?

Procedure if early recurrence is identified

Either:

1. Operate immediately, if suitably trained surgeon is present
2. Refer to Bahir Dar for urgent re-op
3. Refer to another local surgical campaign, if there is capacity and a suitably trained surgeon.

Failure to present at 7-10 follow up

1. Is this due to:
   1. Death
   2. Travelled
   3. Unknown
2. Driver and suture removal nurse to take all possible steps to find patient or arrange for their presentation as soon as possible for suture removal.

Late complications

If patient re-presents between the 7-10 day follow up and the 3 month follow up, record reason and presence/absence of any of the above complications and follow procedure above.

1. **3 month follow up**

Team

Field co-ordinator, driver and assistants

Aims

1. Assess for early recurrence
2. GPS co-ordinates of patient
3. **6 month follow up**

Team

Field Co-ordinator with SR assistance

Driver 1

Assistants

Aims

Full examination and photography

Swabs

1. **One year follow up**

Team

SR

Field co-ordinator

Assistant 5

Driver 1

Aims

Full examination and photography

Questionnaire

Visual acuity

Swabs

1. **18 month follow up**

Team

Field co-ordinator with SR assistance

Driver 1

Assistants

Aims

Full examination and photography

Swabs

1. **Two year follow up**

Team

SR

Field co-ordinator

Assistants

Driver 1

Aims

Full examination and photography

Swabs

**Typical project day protocol (diagram 1)**

Triage patient

Identify suitable areas for different parts of recruitment process

Lecture and info sheet to all patients

Not trachoma

Trachoma

Emergency

Non-emergency

Arrangements for urgent attendance at local eye unit or acute treatment in field if required and possible.

Instructions for attendance at local eye unit and referral letter if required

Not suitable for study, i.e. exclusion criteria

Suitable for study 1

Allocate unique patient identifier

Suitable for study 2

Allocate unique patient identifier

Treat in field as per clinical need and patient choice, including surgery if required

Consent

Consent

Questionnaire

Questionnaire

Measure VA, height, weight

Measure VA, height, weight

Examine patient

Examine patient

Photograph

Photograph

Randomise

Randomise

Surgery

Surgery

Epilation training

Post-op instructions

Post-op instructions

Post-op instructions

Nurse 1, assistants 1,2 + SR

Nurse 1

SR/EO/Nurse 1,2,3 or 4

Nurse 1,2,3 or 4

Nurse 2,3 or 4

Assistants 1+2

SR, Nurse 1, Assistnant 5

Assistant 6

Nurse 2,3,4 assistant 7

Assistant 8

Assistant 6

Nurse 2,3,4 assistant 7

SR

Nurses 2,3,4

Nurses 2,3,4

Assistants 3+4

Nurses 2,3,4

**Appendix A – The project team**

**The team**

SR (Research Fellow)

Co-ordination

Examination and clinical photography

Field co-ordinator (nurse 1)

Trained in:

1. Examination and will then conduct 6 and 18 month follow up.
2. Triage

Nurse 2, 3 and 4 (surgery nurses)

Trachoma surgeons. Technique validated and standardised by Dr Bedri/Dr Abdul

Assistants 1 and 2 (consent field workers)

Trained in:

1. Project explanation/presentation
2. Consent taking
3. Questionnaire administration

Assistants 3 and 4 (Vital statistics field workers)

Trained in measuring

1. Logmar visual acuity
2. Height
3. Weight

Assistant 5 (examination field worker)

Trained in assisting SR with:

1. Examination
2. Photography
3. Microbiological swabbing

Assistant 6 (randomisation field worker)

Trained in how randomisation/masking etc

Assistant 7 (surgical assistant)

Trained in assisting nurses with surgery

Assistant 8 (epilation field worker)

Trained in teaching epilation

Driver 1

Project team driver

Driver 2

Project team driver; suture removal team driver

**Appendix B – Questionnaire and examination protocol and study record form**

**Patient Examination**

1. **General Examination**
   1. **Height in centimetres**
   2. **Weight in kilograms**
   3. **Determine trial eye**

Use pre-designed random eye table

- 1. **Record eye being treated**

1. **Ophthalmic Examination**
   1. **Visual Acuity:**  **EDTRS tumbling E logmar chart at 4m**

a) Best corrected

b) Pinhole vision

To calculate LogMAR visual acuity for 4m:

1. Test to lowest line on which any letter can be read. Complete whole line, even if many mistakes.

2. Take the LogMAR score for the lowest complete line read

3. Add 0.02 x n to this score, where n = the number of letters read from the non-completed line.

N.B

If chart is read at 2m: add 0.3 correction

If chart is read at 1m: add 0.6 correction

**3.2-3.8 Assess Trichiasis**

Assess eyelash position with eye in primary position (looking straight ahead)

- 1. Number of lashes whose point touches globe medial to cornea
  2. Number of lashes whose point touches globe lateral to cornea
  3. Number of lashes whose point touches cornea
  4. Determine grade of trichiasis

| **Trichiasis Grade** | **Definition** |
| --- | --- |
| T 0 | No trichiasis |
| T 1 | Lashes deviated towards the eye, but not touching the globe |
| T 2 | Lashes touching the globe but not rubbing the cornea. |
| T 3 | Lashes constantly rubbing the cornea. |

- 1. Count number of metaplastic lashes right and left eye (*i.e. trichiasis not secondary to entropion*)
  2. Count number of misdirected lashes right and left eye

| Number of metaplastic/misdirected lashes | Right eye | Left eye |
| --- | --- | --- |

- 1. **Count lower lid trichiatic lashes**
  2. **Assess entropion**

1. Assess orientation of the lid margin of the eye in the primary position.

- If necessary, gently raise any excess folds of upper lid skin, *without*, disturbing the position of the upper lid.

*If there is a mixed picture, classify as the worse grade.*

| **Degree of severity** | **Area of entropion** | | |
| --- | --- | --- | --- |
|  | **<50% of lid margin** | **>50% of lid margin** | |
| None | E0 (none) | | |
| Without corneal-lash base contact | E1 (mild) | E2 (moderate) | |
| With corneal-lash base contact | E3 (severe) | | E4 (total) |

None ‘Normal’ lid margin (see glossary) visible

Without corneal-lash base contact Definite inwards rotation of the lid margin, without any lash bases touching the cornea

With corneal-lash base contact Inward rotation of lid margin, with some or all of the lash bases touch the cornea.

**3.10. - 3.12 Assess epilation**

- 1. Is there clinical evidence of epilation? No/yes
  2. How much epilation is there? None, <1/3^rd^, 1/3rd-2/3^rd^, >2/3^rd^
  3. How successful is the epilation? none, successful, <6 lashes, 6+ lashes
  4. **Photograph trichiasis (right and left)**
  5. **Assess plica semilunaris:**

Is the plica semilunaris:

a) Normal: see glossary.

b) Diseased but present. A fold of skin is present, but this is either distorted in shape or scarred down to the underlying conjunctiva.

c) Effaced: the plica semilunaris is absent.

- 1. **Assess for symblepharon (upper or lower): no/yes**

**3.16. – 3.17. Assess for ocular discharge**

- 1. Is there discharge? No/Serous/Purulent/Other (describe)?
  2. **Examine corneal scarring**

If there are more than one corneal scars grade as for worst/most central scar.

a) Grade corneal scarring (see card for diagrammatic representations of corneal scar grades. See appendix F)

C1 Opacity not entering central 4mm

C2a Opacity within central 4mm but not entering within the central 1mm of the cornea. The pupil margin is visible through the opacity.

C2b Opacity within central 4mm but not entering within the central 1mm of the cornea. The pupil margin is not visible through the opacity.

C2c Opacity within central 4mm and entering the central 1mm of the cornea. The pupil margin is visible through the opacity.

C2d Opacity within central 4mm and entering within the central 1mm of the cornea. The pupil margin is not visible through the opacity.

C3 Opacity large enough and dense enough to make whole pupil margin invisible.

C4 Phthisis

- 1. **Photograph cornea**

Photo grading:

1) Size/area

2) Density

i) Mild haziness = mildly obscures iris architecture

ii) Moderate opacity = moderately obscures iris architecture

iii) Severe opacity = iris architecture not visible

If there are >1 opacities, then each should be photo-graded independently.

**Perform bacteriology swab**

- 1. Insert one drop of proxymetacaine
  2. Sweep four times horizontally in the inferior fornix particularly in areas of discharge.
  3. Break swab off into tube containing Stuarts media and seal tube
  4. Label tube
  5. Check labelling done and correct
  6. Record that swab has been performed on the patient record form
  7. Place tube in sample box in the cool box
  8. **Lagophthalmos: no/yes**
  9. **– 3.24. Evert eyelid and continue examination**

**Sequence: 1) Evert with stick end of swab**

**2) Grade**

**3) Photo**

**4) Swab**

- 1. **Assess the presence of conjunctivalisation of the lid margin (assess with upper lid everted)**

CM 0 No conjunctivalisation of the lid margin

CM 1 The muco-cutaneous junction is located anterior to its normal position, but the whole line is still posterior to the line of Meibomian gland orifices.

CM 2 The muco-cutaneous junction is located anterior to the line of the Meibomian gland orifices for less than 50% of the lid.

CM 3 The muco-cutaneous junction is located anterior to the line of the Meibomian gland orifices for greater than 50% of the lid.

- 1. **Examine upper lid follicles**

a ) Determine grade of follicular reaction – examine only zones 2 and 3, ignore follicles in zone 1

F 0 No follicles.
F 1 1-4 follicles.
F 2 5-10 follicles.
F 3 >10 follicles.

- 1. **Examine upper lid papillary hypertrophy**

1. Determine the grade of severity of papillary hypertrophy:

P 0 Absent: normal appearance

P1 Minimal: individual vascular tufts (papillae) prominent, but deep subconjunctival vessels on the tarsus are not obscured.

P2 Moderate: more prominent papillae and normal vessels appear hazy, even when seen by the naked eye.

P3 Pronounced: conjunctiva thickened and opaque, normal vessels on the tarsus are hidden over more than half of the surface.

b) Determine whether papillary reaction is over less than or greater than 50% of the tarsal area.

Papillary inflammation grading

|  | <50% of tarsal area inflamed | >50% of tarsal area inflamed |
| --- | --- | --- |
| None | 0 | 0 |
| Mild | 1 | 2 |
| Moderate | 3 | 4 |
| Severe | 5 | 6 |

To convert to traditional papillary grading system use this table:

| Degree of inflammation /’redness’ | <50% of tarsal area inflamed | >50% of tarsal area inflamed |
| --- | --- | --- |
| None | P0 | P0 |
| Mild | P1 | P1 |
| Moderate | P2 | P2 |
| Severe | P2 | P3 |

**3.24. – 3.25. Conjunctival scarring**

- 1. Conjunctival scarring if no previous surgery Examine upper lid conjunctival scarring (use remainder of swab stick to evert lid)

C0 No scarring on the conjunctiva

C1 Mild: fine scattered scars on the upper tarsal conjunctiva, or scars on the other parts of the conjunctiva

C2 Moderate: more severe scarring but without shortening or distortion of the upper tarsus.

C3 Severe: scarring with distortion of the upper tarsus.

C6 Not applicable

- 1. Conjunctival scarring if previous surgery

SCO No scarring on the conjunctiva

SC1 Surgical line only.

SC2 Surgical line and occasional scattered scars

SC3 Surgical scar with widespread trachomatous scarring but no distortion

SC4 Surgical scar with distortion immediately around the incision line.

SC5 Surgical scar with additional distortion secondary to widespread trachomatous scarring.

SC6 Not applicable

- 1. **Shallowing/loss of the lower fornix: No/yes**
  2. **Photograph tarsal plate**
  3. **Any other examination notes**
  4. **Record if bacteriology swab performed**
  5. **Record bacteriology swab number**
  6. **Perform RNA swab**
  7. Evert upper lid
  8. Sweep swab horizontally four times with a quarter turn of swab after each pass.
  9. Place swab into RNAlater tube and seal tube.
  10. Label tube
  11. Check labelling is correct
  12. Record on patient record form that swab has been collected
  13. Place tube in cool box
  14. On return to BD place tube into fridge over night.
  15. Place in freezer the following morning.
  16. **Record RNA swab number**
  17. **Perform Chlamydial DNA swab**
  18. Evert upper lid
  19. Sweep swab horizontally four times with a quarter turn of swab after each pass.
  20. Place swab into empty tube and seal tube.
  21. Label tube
  22. Check labelling is correct
  23. Record on patient record form that swab has been collected
  24. Place tube in cool box
  25. On return to BD place tube immediately into freezer.
  26. **Record Chlamydial DNA swab number**

**Study** Record Form BASELINE Trial 1 and 2

| Demographic | |
| --- | --- |
| - 1. Study reference number |  |
| - 1. Recruitment Location |  |
| - 1. Recruitment Date |  |
| - 1. First Name |  |
| - 1. Father’s Name |  |
| - 1. Grandfather’s Name |  |
| - 1. Household Head’s Name |  |
| - 1. Husband’s Name (if female + married) |  |
| - 1. Gott |  |
| - 1. Kabele |  |
| - 1. Woredah |  |
| - 1. Telephone number (if possible) |  |
| - 1. Who’s tel is this |  |
| - 1. Other demographic notes |  |

|  | **Answer Options** | **Answer** |
| --- | --- | --- |
| - 1. Sex | 1 = Male  2 = Female |  |
| - 1. Age | Years |  |
| - 1. Date of Birth (Ethiopian) | Date/Month/Year (approx if unknown) |  |
| - 1. Ethnic Group | *Coding to be determined in Ethiopia* |  |
| - 1. Literacy | 0 = Illiterate  1 = Able to read Amharic only  2 = Able to read English only  3 = Able to read other language only (specify)  4 = Able to read English and Amharic/other  5 = Refused to answer |  |

| **General Examination** | | |
| --- | --- | --- |
| - 1. Height |  | |
| - 1. Weight |  | |
| - 1. Trial eye |  | |
| - 1. Eye being treated | 1=Right  2=Left  3=Bilateral |  |

| Ophthalmic Examination (Trial patients only) | | | | | | |
| --- | --- | --- | --- | --- | --- | --- |
|  | **Answer Options** | **Right** | | **Left** | | |
| - 1. Logmar Visual Acuity (4m if possible) | Best Corrected  Pinhole | Line/ Letters | Dist | | Line/ Letters | Dist |
|  |  | / |  | | / |  |
|  |  | / |  | | / |  |
| - 1. No. of medial globe lashes |  |  | | |  | |
| - 1. No. of lateral globe lashes |  |  | | |  | |
| - 1. No. of corneal lashes |  |  | | |  | |
| - 1. Trichiasis grade | T 0,1,2,3 |  | | |  | |
| - 1. Number of metaplastic lashes |  |  | | |  | |
| - 1. Number of misdirected lashes |  |  | | |  | |
| - 1. Lower lid trichiasis | 0 = No  1 = Yes |  | | |  | |
| - 1. Entropion grade | E 0,1,2,3,4 |  | | |  | |
| - 1. Epilation | 0 = No  1 = Yes |  | | |  | |
| - 1. Epilation; how much | 0 = none  1 = <1/3rd  2 = 1/3^rd^-2/3^rd^  3 = >2/3rd |  | | |  | |
| - 1. Epilation; how successful | 0 = no epilation  1 = successful  2 = <6 lashes  3 = 6+ lashes |  | | |  | |
| - 1. Photo TT | 0 = No  1 = Yes |  | | |  | |
| - 1. Plica semilunaris | 0 = Normal  1 = Diseased but present  2 = Effaced |  | | |  | |
| - 1. Symblepharon (upper or lower) | 0 = No  1 = Yes |  | | |  | |
| - 1. Ocular discharge | 0 = No  1 = Serous (watery)  2 = Purulent  3 = Other (describe) |  | | |  | |
| - 1. Corneal scar grade | CO 1,2a,2b,2c,2d,3,4 |  | | |  | |
| - 1. Photo cornea | 0 = No  1 = Yes |  | | |  | |
| **Perform bacteriology swab – see below for coding** | | | | | | |
| - 1. Lagophthalmos | 0 = No  1 = Yes |  | | |  | |
| - 1. Conjunctivilisation of margin grade | CM 0,1,2,3 |  | | |  | |
| - 1. Follicle grade | F 0,1,2,3 |  | | |  | |
| - 1. Papillary grade | P 0,1,2,3,4,5,6 |  | | |  | |
| - 1. Conj scar grade | C 0,1,2,3,6(n/a) |  | | |  | |
| - 1. Conj scar grade if prev surgery | SC 0,1,2,3,4,5,6(n/a) |  | | |  | |
| - 1. Shallowing/loss of lower fornix | 0 = No  1 = Yes |  | | |  | |
| - 1. Tarsal plate photo | 0 = No  1 = Yes |  | | |  | |
| - 1. Other examination Notes | ***Right*** | ***Left*** | | | | |
| - 1. Swab conj bacteria | 0 = No  1 = Yes |  | | |  | |
| - 1. Bacteriology Swab No. |  |  | | |  | |
| - 1. Swab RNA | 0 = No  1 = Yes |  | | |  | |
| - 1. RNA Swab Number |  |  | | |  | |
| - 1. Swab DNA | 0 = No  1 = Yes |  | | |  | |
| - 1. DNA Swab Number |  |  | | |  | |
| - 1. Shirmer’s 1 (pre anaesthetic) | mm in 5 minutes |  | | |  | |
| - 1. Shirmer’s 2 (post anaesthetic) | Mm in 5 minutes |  | | |  | |

| Ophthalmic Questionnaire (Trial patients only) | | | | | |
| --- | --- | --- | --- | --- | --- |
|  | | **Answer Options** | **Right** | | **Left** |
| - 1. Have you ever had eye surgery | | 1 = No  2 = Trachoma surgery  3 = Non trachoma surgery (specify what surgery) |  | |  |
| - 1. If yes, what surgery | | Free text |  | |  |
| - 1. Do you have any eye pain? | | 0 = No  1 = Yes |  | |  |
| - 1. If you do have eye pain, how often do you experience this? | | 0 = Never  1 = Once a week  2 = Once a day  3 = Several times a day  4 = Constantly |  | |  |
| - 1. Do you eyes water | | 0 = Never  1 = Occasionally  2 = Often |  | |  |
| - 1. During a typical day in the past week, how often did your eyes feel dry (so that you wanted to put water or other lubricant in them) | | 0 = Never 1 = Infrequently 2 = Frequently 3 = Constantly 4 = Not sure |  | |  |
| - 1. Do you epilate? | | 0 = No  1 = Yes |  | |  |
| - 1. How often do you epilate? | | 0 = Never  1 = More than once a week  2 = once/week to once/month  3 = Less than once a month |  | |  |
| - 1. When did you last epilate? | | 0 = Never  1 = Within last week  2 = 1 week to 1 month ago  3 = Greater than 1 month ago |  | |  |
| - 1. Who does the epilating? | | 0 = never  1 = You  2 = A friend or relative  3 = Health care worker |  | |  |
| - 1. What do you epilate with | | 0 = Don’t epilate  1 = Locally made forceps  2 = Machine made forceps  3 = Burning  4 = Cutting |  | |  |
| - 1. Has a health-worker previously told you that need eyelid surgery | | 0 = No  1 = Yes |  | |  |
| - 1. When was the last time you were offered surgery? | | 0 = Never  1 = Within last year  2 = >1 year ago |  | |  |
| - 1. If surgery has been recommended or offered to you in the past, but you have not received or accepted the surgery, what are your main reasons for this? *(list as many as appropriate)* | 0 = Never offered surgery (i.e. n/a) | | | *Yes No* | |
|  | 1 = No symptoms | | | *Yes No* | |
|  | 2 = Symptoms present, but didn’t know treatment needed | | | *Yes No* | |
|  | 3 = Cost | | | *Yes No* | |
|  | 4 = No one to accompany | | | *Yes No* | |
|  | 5 = Lack of time | | | *Yes No* | |
|  | 6 = Fear of the operation | | | *Yes No* | |
|  | 7 = Resistance of family | | | *Yes No* | |
|  | 8 = Transport difficulties | | | *Yes No* | |
|  | 9 = Don’t know where to get it | | | *Yes No* | |
|  | 10 = Other (specify reason) | | | *Yes No* | |
| Of the above reasons which is:   - 1. the most important*:* | |  | | | |
| - 1. the 2^nd^ most important | |  | | | |

**I certify that all the demographic questions are completed_______________ __ Date_________**

**I certify that all the questionnaire questions are completed______________ Date_________**

**I certify that all the examination findings are recorded__________________ Date_________**

**I certify that all the answers are accurately entered on the database_______ Date_________**

**I certify that all the answers are accurately entered on the database_______ Date_________**

**Appendix C – Randomisation procedure**

**Randomisation trial 1**

- Blocked randomisation.
- Suggest block sizes of 4 as any more and there is a risk of imbalance on a day where little surgery is performed.
- Helen Weiss/appropriately delegated person to finalise block size. No-one in the field including Saul to know it.
- Stratify for three (number to be confirmed) surgeons and each surgeon will have their own separate randomisation sequence
- S=suture trial
- E=epilation trial

**A. Designing of randomisation code master sheet – to be carried out by Helen Weiss or person delegated by Helen.**

1. Surgeon 1’s randomisation envelope sequence will be marked as follows S1/0001-S1/1000
2. Surgeon 2’s randomisation envelope sequence will be marked as follows S2/0001-S2/1000
3. Surgeon 3’s randomisation envelope sequence will be marked as follows S3/0001-S3/1000
4. Possible randomization procedure, e.g. for blocks of 4:
   1. List the 6 possible combinations of vicryl and silk (i.e. [1]VVSS, [2]:VSVS, [3]:VSSV etc). These are combinations 1-6.
   2. Identify a random starting point on the random numbers table.
   3. Read across the table until a number between 1 and 6 is seen. This number instructs which of the combinations to use.
   4. Write down this combination (of 4 suture allocations) on the master code list for sequential patients.
   5. Continue determining series of 4 allocations until all 3000 unique identifiers have a suture assigned to them
5. Helen to keep one copy of the master code sheet. 2 further copies to be sent to people not involved in the trial for safe keeping. A 4^th^ copy to be sent to a person in Ethiopia independent of the trial who will transfer code to individual envelopes.

Master list S1

| **Surgeon 1** | | **Surgeon 2** | | **Surgeon 3** | |
| --- | --- | --- | --- | --- | --- |
| **U.I.** | **Suture** | **U.I.** | **Suture** | **U.I.** | **Suture** |
| S1/0001 | e.g vicryl | S2/0001 | e.g. silk | S3/0001 | e.g. vicryl |
| S1/0002 | e.g. silk | S2/0002 |  | S3/0002 |  |
| Etc |  | etc |  | etc |  |

**B. Making up surgeon instruction envelopes from code sheet – to be carried out by a person independent of the trial.**

1. Prepare three boxes that can each hold 1000 envelopes. Mark the boxes Surgeon 1, 2 and 3.
2. Print S1/0001-S1/1000 on to 1000 white envelopes for surgeon 1
3. Print S2/0001-S2/1000 on to 1000 another colour envelopes for surgeon 2
4. Print S3/0001-S3/1000 on to 1000 a third colour envelopes for surgeon 3
5. Following the master code sheet do the following:
   1. Print the randomisation number and the allocated suture instruction for this number on a piece of paper and place in the envelope.
   2. Do NOT seal the envelope.
6. In batches of 10 double check that each envelope contains the correct card. Then seal.

**C. In the field – to be performed by dedicated trained randomisation field worker**

1. Randomisation field worker will be working in location not visible to other project team members.
2. After consent and examination, the patient is sat in a waiting area.
3. When a surgeon is available for operating, the surgeon finds the randomisation field worker and together they take the patient through to the theatre.
4. Again, together they take the next envelope from that surgeon’s pile and open it. They will open the envelope and record the instruction on masterlist S2 (see below). This will be recorded on two copies of masterlist two, with one copy being kept by the randomisation field worker and the second copy being given to a person independent of the trial. A new masterlist S2 page will be started on each trial day.
5. Field worker will take 3 (if unilateral surgery required) or 6 (if bilateral) of the correct sutures from the suture store and give to the surgeon. They will ask the surgeon to double check that this is the correct suture. They will both sign masterlist 2 to confirm that correct sutures have been taken

Master list S2 – to be recorded in a book 1 ‘trial 1 suture choice book’

| Date | Study Number | Name | Patient unique identifier | Randomisation Envelope | Sutures Used | Field worker signature | Surgeon signature |
| --- | --- | --- | --- | --- | --- | --- | --- |
|  | 1 | XX |  | S1/0001 | V |  |  |
|  | 2 | Xx |  | S2/0001 | V |  |  |
|  | 3 | Xx |  | S3/0001 | S |  |  |
|  | 4 | Xx |  | S1/0002 | V |  |  |
|  | 5 | Xx |  | S3/0002 | S |  |  |
|  | 6 | Xx |  | S2/0002 | S |  |  |

1. The surgeon will record the details of the operation in masterlist S3 (see below), to record the suture type, complications and whether suture removal should be performed in 7-10 days. This will be recorded on two copies of masterlist S3 (2 logbooks).
2. The randomisation field worker will photocopy masterlist S3 at the end of each day and highlight all patients requiring suture removal. This highlighted list will be given to the suture removal nurse.

Masterlist S3 – to be recorded in trial 1 ‘surgeons log-book’

| Unique identifier | Name | Date of surgery | Suture type (vicryl or silk) | Complications | Surgeon number | Surgeon signature | Date for suture removal | Sutures successfully removed  (yes or no) | Signature of suture remover |
| --- | --- | --- | --- | --- | --- | --- | --- | --- | --- |
|  |  |  |  |  |  |  |  |  |  |
|  |  |  |  |  |  |  |  |  |  |
|  |  |  |  |  |  |  |  |  |  |

**Breaking the code**

Only to be done for an individual patient by Tanzi Edwards, on the instructions of the trial steering committee.

**Appendix D – The Trabut Surgical Technique**

Summary of method (as per Final Assessment of Trichiasis Surgeons, WHO 2005)

1. Refer to the WHO yellow manual for aseptic technique and administration of local anaesthetic.

2. Application of traction suture. The lid should not be everted at the start. Approximately 3 mm from the upper lid margin, insert the needle with suture through the skin and orbicularis, starting either laterally or medially. Take two large bites, about 5 mm in length, with a similar space in between. There should be a loop in the middle.

3. Everting the lid and keeping it in position. Sling the suture loop into a Trabut entropion plate or Wilde’s entropion forceps, pulling the two suture ends and at the same time everting the lid. Use the forceps as a fulcrum as the lid is everted. The lid is kept in the everted position by clamping

the suture to the drape using the haemostat forceps.

4. Incision of tarso-conjunctiva. Using the blade, scratch an incision along the Arlt’s line (2–3 mm from the margin) and deepen the cut until the orbicularis muscle is reached (through the tarsal plate). Extend the incision over the whole length of the tarsal plate, ending just before the

lachrymal punctum medially and laterally at the canthus. Place a tissue forceps on the proximal tarsal conjunctiva to hold the incised tarso-conjunctiva, and complete the incision with scissors.

5. Blunt dissection of the tarso-conjunctiva. Pick up the tarso-conjunctiva with tissue forceps. Insert closed scissors and spread them, dissecting the tarso-conjunctiva from the overlying orbicularis muscle. The dissection should extend for approximately 8 mm.

6. Suturing to achieve eversion of the distal fragment of the lid margin. Use mattress sutures, taking 1-mm bites of tarsal conjunctiva and half the thickness of the tarsal plate, passing the needle under the distal tarsal conjunctiva and emerging through the skin about 3 mm above the

lid margin. A minimum of three or four evenly spaced mattress sutures are to be applied. To finish, all the sutures are pulled up together so as to bury the proximal fragment edge of the tarso-conjunctiva under the distal fragment. Starting in the middle, tie the sutures snugly with three single knots, and cut 3 mm above the knot.

7. Remove the traction suture and the Wilde’s entropion forceps or Trabut plate. Apply topical antibiotic and dressing as described for BLTR.

**Appendix E – The sterilisation of instruments (as per The WHO Manual for Trichiasis Surgery for Trachoma)**

Sterilization is defined as the destruction of all viruses, bacteria and spores. High-level disinfection is defined as the destruction of all viruses and bacteria, but spores may survive if initially present in large numbers.

(a) Sterilization by Steam

Steam sterilization is performed under pressure for at least 15 minutes after the load reaches a temperature of 121 degrees Centigraded (250 Fahrenheit), at a pressure of 1 atmosphere above atmospheric pressure (101 kPa, 15 lb/sq.in.) and after water vapour saturation.

(c) Sterilization by Dry Heat

Sterilization in an electric or gas oven is achieved after two hours at 170 degrees Centigrade (340 degrees Fahrenheit), allowing additional time prior to this for the load to equilibrate at that temperature.

(d) High-level disinfection by boiling

The instruments are boiled in water, for at LEAST 20 MINUTES

(d) High-level disinfection by soaking in Glutaraldehyde

Glutaraldehyde is obtained as a 2% aqueous solution to which a powder or liquid buffer (supplied with it) is added to render the solution active. Once activated, the solution should not be kept for more than two weeks. Instruments should be thoroughly cleaned before soaking, to remove clotted blood or tissue. They are then completely immersed for AT LEAST 30 MINUTES. After soaking, the instruments are only handled with sterile gloves, towels, or forceps. They are rinsed with sterile saline or water before use. This technique is not acceptable for the needles and syringes used for injection, which must therefore be disposable

**Appendix F – Diagrammatic Representation of Corneal Scar Grading**

**Glossary**

Dystichiasis

A congenital aberrant row of lashes.

Entropion

Inwards rotation of the lid margin.

Lid margin

The free margin of the eyelid. In the normal eye this is approximately 2mm thick and square in cross section, except in the medial one-sixth (medial to the punctum) where it is rounded. The outer boundary of the lid margin is the lash base line of the inner most row of eyelashes. The lid margin is covered in skin continuous with the outer lid and contains meibomian gland openings in an orderly row. The inner margin is where the skin ends, just prior to where the lid touches the cornea in primary position and conjunctiva begins.

Metaplastic lashes

Acquired aberrantly located eyelashes. These often arise from meibomian gland orifices.

Misdirected lashes

Lashes that point in an abnormal direction, but whose base is in a normal position.

Plica Semilunaris

A semi-lunar fold of conjunctiva lying on the lateral side and partly underneath of the caruncle. In the normal eye, the lateral margin is free and concave. Beneath the fold is a small space about 2mm deep when the eye is looking medially. When the eye is looking laterally, the space practically disappears.
